# Diseases of the Throat, Nose, and Ear

**Published:** 1916-12

**Authors:** 


**Diseases of the Throat, Nose, and Ear.** By W. G. Porter, M.B. Second Edition. Pp. xvi. 280. Illustrated. Bristol; John Wright & Sons Ltd. 1916. Price 7s. 6d. net.

A special interest attaches to this edition, as it is brought out during the absence of the author, who is on service with the British Expeditionary Forces, and hence has been revised by Dr. Peter McBride, who also, in the introductory preface, expresses his view that the work admirably fulfils its object, and in this we fully concur. Dr. Porter's handy treatise not only preserves throughout the due sense of proportion, omitting little that one ought to find in a faithful and up-to-date picture of modern oto-laryngology for the general practitioner and senior student, but, what is perhaps more difficult to attain, not going beyond its purposed limitations. The matter of the text is accurately and clearly set forth; the illustrations, both coloured and black and white, are typical and plentiful without being so lavish as to confuse by unnecessary multiplication. It is a text-book which thoroughly deserves its excellent reputation, and reflects the highest credit on author and producer alike.

